# Improving Schwann Cell Differentiation from Human Adipose Stem Cells with Metabolic Glycoengineering

**DOI:** 10.3390/cells12081190

**Published:** 2023-04-19

**Authors:** Jian Du, Zihui Wang, Xiao Liu, Cecilia Hu, Kevin J. Yarema, Xiaofeng Jia

**Affiliations:** 1 Department of Neurosurgery, University of Maryland School of Medicine, 10 South Pine Street, MST 823, Baltimore, MD 21201, USA; 2 Department of Biomedical Engineering, The Johns Hopkins School of Medicine, Baltimore, MD 21205, USA; 3 Translational Cell and Tissue Engineering Center, The Johns Hopkins School of Medicine, Baltimore, MD 21231, USA; 4 Department of Orthopedics, University of Maryland School of Medicine, Baltimore, MD 21201, USA; 5 Department of Anatomy and Neurobiology, University of Maryland School of Medicine, Baltimore, MD 21201, USA

**Keywords:** metabolic glycoengineering, human adipose stem cells, cell differentiation, Schwann cell, nerve regeneration

## Abstract

Schwann cells (SCs) are myelinating cells that promote peripheral nerve regeneration. When nerve lesions form, SCs are destroyed, ultimately hindering nerve repair. The difficulty in treating nerve repair is exacerbated due to SC’s limited and slow expansion capacity. Therapeutic use of adipose-derived stem cells (ASCs) is emerging in combating peripheral nerve injury due to these cells’ SC differentiation capability and can be harvested easily in large numbers. Despite ASC’s therapeutic potential, their transdifferentiation period typically takes more than two weeks. In this study, we demonstrate that metabolic glycoengineering (MGE) technology enhances ASC differentiation into SCs. Specifically, the sugar analog Ac_5_ManNTProp (TProp), which modulates cell surface sialylation, significantly improved ASC differentiation with upregulated SC protein S100β and p75NGFR expression and elevated the neurotrophic factors nerve growth factor beta (NGFβ) and glial cell-line-derived neurotrophic factor (GDNF). TProp treatment remarkably reduced the SC transdifferentiation period from about two weeks to two days in vitro, which has the potential to improve neuronal regeneration and facilitate future use of ASCs in regenerative medicine.

## 1. Introduction

Schwann cells (SCs) are the critical glial cells due to their roles in the development, maintenance, function, and regeneration of nerves in the peripheral nervous system [1,2,3]. SCs are able to improve nerve regeneration after nerve injury through decreased expression of myelin-related protein and increased expression of cell adhesion molecules, neurotrophic factors, and cytokines [4,5,6,7]. These changes facilitate axonal regeneration by guiding distal nerve stump and basal lamina growth. In addition, axonal regeneration requires renewed axon–Schwann cell interactions, which leads to axonal remyelination and restoration of the physiologic nerve function [8,9]. Unlike nerves from the central nervous system, peripheral nerves are able to regenerate following injury, but often inadequately [10]. When nerve lesions form, SCs are damaged, ultimately leading to poor functional recovery in patients. The difficulty in treating peripheral nerve repair is exacerbated due to the SCs’ limited and slow expansion capacity [11,12].

Adipose-derived stem cells (ASCs) that have been differentiated towards a Schwann-like cell phenotype can act as a therapeutic alternative [13,14]. ASCs differ from SCs in that they can be harvested in large numbers using minimally invasive techniques [15,16,17], and have been widely reported in augmenting peripheral nerve regeneration [18,19]. Prior research has established that morphological changes to ASC-derived Schwann-like cells (dASCs) are dependent on a cocktail of extrinsic factors, such as forskolin, and several neurotrophic factors for maintenance [13]. These extrinsic factors cause the cells to exhibit elongated spindles and to express the glial markers, GFAP, S100, and p75, characteristic of SCs [20,21,22] and myelin proteins and structures (when in co-culture with neurons) [23]. Similarly, when implanted in nerve conduits to connect the ends of a murine peripheral nerve gap in vivo, dASCs promote nerve regeneration through SC-like functions by reducing muscle atrophy, increasing nerve conduction velocity and myelination rates, as well as inducing significant improvement in the restoration of function when compared to nerve conduits without exogenously added cells [23,24,25]. Despite these many promising benefits, barriers to using dASCs therapeutically for peripheral nerve repair include a differentiation process that typically takes at least two weeks [13]. During this extended induction period, many ASCs undergo apoptosis, reducing SC yield [23,26] and significantly hindering clinical translation efforts.

**Figure 1:**
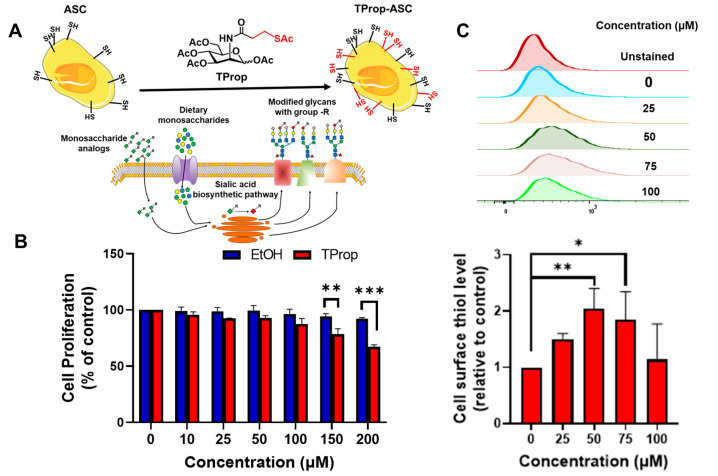
(**A**) Overview of MGE: ASCs are incubated with TProp resulting in incorporation into cell-surface displayed sialoglycans, thereby increasing the cell surface expression of thiol groups. (**B**) Cellular metabolic activity was determined by the MTT metabolic assay after 3 days of incubation with EtOH (vehicle control) or Ac_5_ManNTProp (TProp). All results were normalized to the vehicle control samples. Dose-response experiments showed that TProp slowed the growth of the cells at concentrations over 100 μM. Student’s *t*-test statistical analyses were used to compare differences between vehicle control and exposed conditions. Mean ± SEM; *n* = 3 independent experiments; ** *p* < 0.01, *** *p* < 0.001. (**C**) Cell surface thiols were quantified by flow cytometry with ethanol control samples arbitrarily set to a value of 1.0. Mean ± SEM; *n* = 3 independent experiments; * *p* < 0.05, ** *p* < 0.01, one-way ANOVA with Tukey’s post-test. These results indicated the optimized concentration of TProp was 50 µM, a non-cytotoxic level that maximized cell surface; accordingly, 50 µM was used for subsequent experiments in this study.

In this study, we sought to improve ASC transdifferentiation into SCs via metabolic glycoengineering (MGE, Figure 1A). MGE is a strategy where non-natural hexosamines with modified N-acyl groups are supplied to cells or living animals; when appropriately designed, the sugar analogs intercept biosynthetic pathways and are incorporated into cell surface glycans [27,28,29]. Recently, our team has demonstrated “second generation” thiolated analog Ac_5_ManNTProp (TProp) [30] that replaces natural cell surface sialic acids with their thiolated counterparts, thereby improving the differentiation of human neural stem cells (hNSCs) as well as their adhesion to extracellular matrix components [31]. In the current study we extend the repertoire of beneficial therapeutic activities made possible through thiol-modified MGE analogs by demonstrating the ability of TProp to facilitate SC differentiation from ASCs. Overall, these studies showed that MGE more efficiently induces differentiation of ASCs to SCs compared to conventional methods currently available.

## 2. Materials and Methods

### 2.1. Metabolic Activity

Human adipose stem cells (ASCs) were a gift from Dr. Warren Grayson’s laboratory at Johns Hopkins University. Human subcutaneous adipose tissue was obtained in the form of lipoaspirate from a healthy 47-year-old Caucasian female donor undergoing elective liposuction surgery, with written informed consent, and with the Johns Hopkins University Institutional Review Board approval. ASCs were isolated as previously described [6,32,33] and their characteristics have been reported previously [34]. These cells were isolated from the lipoaspirate tissue of an adult female patient with approval from the Johns Hopkins Medicine Institutional Review Board. To access the impact of TProp on ASC proliferation, 1.0 × 10^4^ ASCs per well were seeded in a 96-well plate and placed in a water-saturated incubator at 5.0% CO_2_. The maintenance medium used was DMEM supplemented with 10% (*v*/*v*) FBS, 5 ng/mL basic fibroblast growth factor (bFGF; PeproTech), and 100 U/mL penicillin/100 μg/mL streptomycin (P/S, Invitrogen, Waltham, MA, USA) [35,36]. After 24 h, either TProp or an equal volume of the solvent vehicle, ethanol (to a maximum of 0.1%, *v*/*v*) were added to the wells at concentrations between 0–200 μM. After 3 days, an MTT assay was performed as described in our previous publication [30] and the absorbances were read at 570 nm with 630 nm as the reference wavelength by using a multiwell plate reader.

### 2.2. Flow Cytometry

Cell surface thiol (CST) display was measured by flow cytometry, as described in our previous publications [37,38,39]. To quantify CSTs, ASCs were incubated with TProp at concentrations of up to 100 µM. Cells were harvested after 3 d and washed twice with PBS. The cells then were resuspended with 5.0 mM (+)-biotinyl-3-maleimidopropionamidyl-3,6-dioxaoctane-diamine (MB; Pierce Biotechnology, Waltham, MA, USA) in PBS at room temperature. After 1.0 h cells were centrifuged and washed three times with ice-cold avidin staining buffer (PBS containing 5.0% FBS and 0.1% NaN_3_). The cell suspension was next mixed with FITC-labeled avidin (Sigma, St. Louis, MO, USA) for 15 min on ice. Finally, cells were washed three times with avidin staining buffer and analyzed by flow cytometry on a BD Canto II instrument. For each measurement, ~10^4^ cells were counted in triplicate from three replicate samples.

### 2.3. ASC Schwann Cell Differentiation

ASCs of passages three to five with 5 × 10^4^ cells were plated into experimental and control wells in a 24-well plate. After 2 days in the maintenance medium, the culture media in the experimental groups were replaced with the medium previously reported to induce the differentiation of ASCs into Schwann-like cells [13]. Briefly, ASCs were first incubated with 1.0 mM β-mercaptoethanol (BME; Sigma-Aldrich, St. Louis, MO, USA) in DMEM medium for 24 h followed by the addition of 35 ng/mL all-trans-retinoic acid (Sigma-Aldrich, St. Louis, MO, USA) in DMEM medium for 72 h. After this, cells were incubated in SC-conditioned DMEM medium containing 14 µM forskolin (Sigma-Aldrich, St. Louis, MO, USA), 5 ng/mL platelet-derived growth factor-AA (PDGF; PeproTech, Rocky Hill, NJ, USA), 10 ng/mL bFGF, and 200 ng/mL recombinant human heregulin-β1 (HRG; PeproTech, Rocky Hill, NJ, USA) with or without 50 μM TProp. Media were changed every other day. For the TProp group, analog was replenished at each change of media.

### 2.4. Identification of Schwann-Like Cells by Immunofluorescence

Immunocytochemical assessment of ASC markers was performed after the ASCs were cultured in the SC-conditioned medium for either 2 or 14 days. After culturing, the cells were fixed at room temperature in 4% (*w*/*v*) paraformaldehyde for 15 min and then blocked with 5% goat serum for 1.0 h. After the time elapsed, antibodies with specificity for S100 calcium-binding protein β (S100β) and p75 neurotrophin receptor (p75), both from Santa Cruz Biotechnology, Inc. (Dallas, TX, USA), and beta-catenin (β-catenin, Sigma, St. Louis, MO) were added and incubated at 4 °C overnight. The secondary antibodies goat anti-rabbit Cy3- and donkey anti-mouse FITC-conjugated from Fisher Scientific (Hampton, NH, USA ) were added for 1.0 h at room temperature. In addition, cell nuclei were stained with DAPI (Sigma-Aldrich, St. Louis, MO, USA). Fluorescence microscope imaging (Leica DMi8 microscope, Leica Microsystems, Wetzlar, Germany) was then performed on the stained cells. The percentages of S100 and p75 positive cells were calculated by using ImageJ (v 1.70, NIH, Bethesda, MD, USA) Cell Counter plugin. The number of S100 and p75 positive cells was determined by manually counting the number of cells displaying biomarker protein immuno-reactivity. DAPI-positive cells that stained bright blue were automatically counted and designated as the total cell number. Their ratio was presented as the percentage. The positive area (area of β-catenin positive cells per total cell area) and mean intensity of β-catenin positive cells were automatically calculated by using ImageJ (v 1.70, NIH, Bethesda, MD, USA). Three independent experiments were performed. At least three random images of each sample were analyzed for statistics.

### 2.5. Detection of Secreted Nerve Growth Factor Beta (NGFβ) and Glial Cell Line-Derived Neurotrophic Factor (GDNF) by Enzyme-Linked Immunosorbent Assay (ELISA)

During the differentiation of ASCs to Schwann-like cells, their media were collected at day 2 and day 14, and centrifuged at 300× *g* for 10 min at 4 °C. The concentrations of NGFβ and GDNF in the supernatant from each group were measured via ELISA using human NGFβ and human GDNF ELISA Kit (Boster Bio, Pleasanton, CA, USA). The absorbances of each well were recorded using a spectrophotometer with a reference wavelength of 450 nm. The concentrations of NGFβ and GDNF were determined using a standard curve derived from authentic samples of these proteins. All measurements were repeated three times from independent technical replicates.

### 2.6. mRNA Extraction and RT-qPCR Analysis

Transcript quantification began by isolating mRNA from the test cells using the RNeasy Mini Kit (Qiagen, Germantown, MD, USA) followed by cDNA synthesis using a cDNA Reverse Transcription Kit (Applied Biosystems, Waltham, MA, USA). Real-time quantitative PCR (RT-qPCR) was performed using the QuantStudio 3 Real-Time PCR System (Thermo Fisher, Hampton, NH, USA). The primers used were forward 5′-GAAGAAATCCGAACTGAAGGAGC-3′ and reverse 5′-TCCTGGAAGTCACATTCGCCGT-3′ (S-100β), forward 5′-CATCACCTGGAGGACTTCTACC-3′ and reverse 5′-CAGTGTACTGGATGCTCTTCAGG-3′ (NCAM, neural cell adhesion molecule), forward 5′-CTGGAGAGGAAGATTGAGTCGC-3′ and reverse 5′-ACGTCAAGCTCCACATGGACCT-3′ (GFAP, glial fibrillary acidic protein), 5′-GGCAATGGACACGCAACTGATC-3′ and reverse 5′-TGATCGACAGGATCATGGTGGC-3′ (PMP22, peripheral myelin protein 22), forward 5′-CTATCCTGGCTGTGCTGCTCTT-3′ and reverse 5′-ACTCACTGGACCAGAAGGAGCA-3′ (P0, myelin protein zero), and forward 5′-GGAGCGTGGCTACTCTTTTG-3′ and reverse 5′-GGCTGGAAGAGTGTCTCAGG-3′ (GAPDH). The first stage of RT-qPCR involved one cycle of reverse transcription for 30 min at 50 °C. The second stage involved one cycle of predegeneration for 10 min at 95 °C. The third stage involved 40 cyclic reactions at 95 °C for 10 min followed by 60 °C for 30 s. The fourth stage involved one default melting cycle. Relative mRNA expression was then calculated using the 2^−∆∆Cq^ method and the data were normalized using GAPDH mRNA expression.

### 2.7. Statistical Analysis

Statistical differences between the two groups were compared using the Student’s *t*-test. One-way analysis of variance followed by a multiple comparison Tukey’s post-test was used for the multiple groups’ statistical analyses. Each experiment was carried out at least three times, and the data were reported as mean ± SEM. A significant difference was considered by *p* < 0.05.

## 3. Results

### 3.1. Metabolic Impact of Thiol-Modified ManNAc Analogs in ASCs

To determine the optimal concentration of the ManNAc analog TProp, we tested its impact on metabolic viability in ASCs at various concentrations using MTT assays. Ethanol was used as the solvent control to establish a baseline (for context, in our past experience, the small concentrations of ethanol used as a delivery vehicle for MGE analogs have not affected biological activity [30]). Following 3 days of treatment with TProp, ASCs showed a moderate decrease in cell proliferation as analog concentration increased from 0 to 100 µΜ (Figure 1B). There was only a slight decrease in cell proliferation (7.2%) at concentrations up to 50 μM, with a decrease in viability from 92.8% to 87.5% as the analog concentration increased from 50 μM to 100 μM and a further decrease to 78.5% and 67.3% at 150 and 200 μM, respectively. The flow cytometry study (Figure 1C) indicated that the maximal display of cell surface thiol groups in cells treated with TProp peaked at 50 µM of this analog, approximately doubling their expression levels compared to the ethanol-treated control cells. Thus, this optimized concentration of 50 µM TProp was used for the remainder of the experiments reported in this study.

### 3.2. Differentiation of ASCs to a Schwann Cell Phenotype

**Figure 2:**
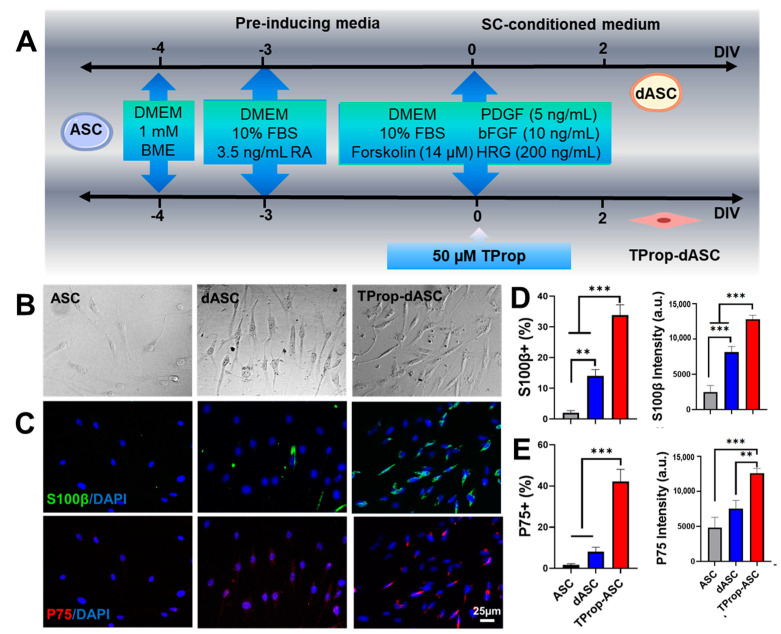
Characterization and comparison of Schwann-cell-like differentiation from ASCs with and without TProp after two days of treatment. (**A**) Schematic of the protocol used to differentiate ASCs into SC-like cells. (**B**) Optical microscopy images showed the flattened morphology in undifferentiated ASCs (ASC) and differentiated ASCs (dASC), whereas a spindle-shaped Schwann cell morphology was observed in the differentiated ASCs treated with 50 µM TProp (TProp-dASC). (**C**) Immunofluorescence staining indicated higher levels of S100 (green) and P75 (red) protein expression in the TProp-dASCs. The scale bar is 25 μm. Quantitative analysis based on percentage of positively stained cells and intensity of S100 (**D**) and P75 (**E**). Data represented mean ± SEM (*n* = 3); ** *p* < 0.01, and *** *p* < 0.001, one-way ANOVA with Tukey’s post-test.

The ASC differentiation procedure is outlined in Figure 2A. The morphologies and biomarkers of differentiated ASCs were evaluated after 2-day and 2-week differentiation periods. Optical microscopy showed that ASCs maintained their original flattened morphology whereas TProp-dASCs developed a spindle-shaped cell morphology similar to genuine SCs (Figure 2B). Immunofluorescence staining (Figure 2C) after 2 days of incubation in the maintenance medium showed negligible S100 (2.001 ± 0.7413% positive cell percentage, 2485 ± 930.6 positive intensity) and P75 protein expression (1.666 ± 0.6158 and 4832 ± 1469) in ASCs whereas cells cultured in the differentiation media (dASC) expressed both proteins (14.02 ± 2.080%, 81,471 ± 795.8 S100 and 8.099 ± 2.169%, 7561 ± 1143 P75 positivity) at measurable levels (Figure 2D,E). When TProp was introduced into the differentiation process, cells expressed higher levels of S100β (33.80 ± 3.385%, *p* < 0.001; 12,810 ± 545.1, *p* < 0.001) and P75 (42.19 ± 5.890%, *p* < 0.001; 12,581 ± 706.1, *p* < 0.01) than those from the dASC group, indicating that TProp boosted cell differentiation in two days.

**Figure 3:**
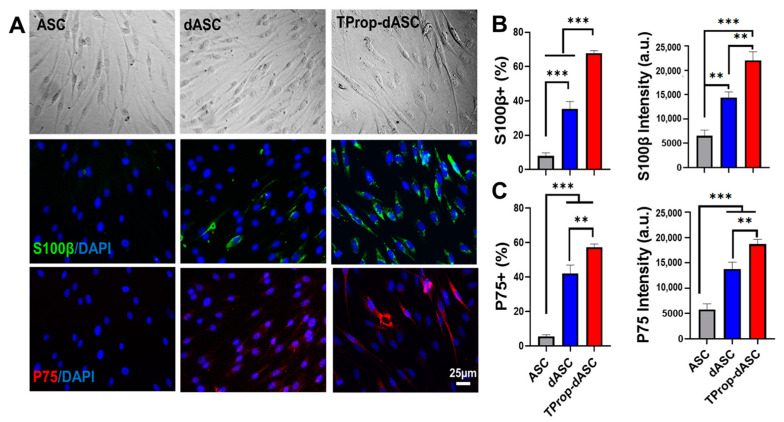
Characterization and comparison of Schwann-cell-like differentiation from ASCs with and without TProp after fourteen days of treatment. (**A**) Bright field images and immunofluorescence staining indicated the different levels of S100 (green) and P75 (red) protein expression in undifferentiated ASCs (ASC), differentiated ASCs (dASC), and differentiated ASCs treated with 50 µM TProp (TProp-dASC). The scale bar is 25 μm. Quantitative analysis of positive cell percentage and intensity of S100 (**B**) and P75 (**C**). Data represented mean ± SEM (*n* = 3); ** *p* < 0.01, and *** *p* < 0.001, one-way ANOVA with Tukey’s post-test.

With the extension of the induction period to 2 weeks, the rate of S100β-positive cells percentage and their protein expression increased (Figure 3A), 8.029 ± 1.752, 35.34 ± 4.301, and 67.84 ± 1.376%; 6531 ± 1161, 14,378 ± 1175, and 22,013 ± 1795 for ASC, dASC, and TProp-dASC groups, respectively (Figure 3B). Both the dASC (*p* < 0.01) and TProp-dASC (*p* < 0.001) groups expressed significantly higher S100 levels than the ASC group, and furthermore, the TProp-dASC group had a higher S100β level (*p* < 0.01) than the dASC group. Quantification of P75 positive cell percentage and expression intensity (Figure 3C) confirmed the SC differentiation. The TProp-dASC (57.37 ± 1.796%, *p* < 0.001; 18,701 ± 943, *p* < 0.001) and dASC (42.17 ± 4.826%, *p* < 0.01; 13,763 ± 1380, *p* < 0.001) groups showed significantly higher P75 positivity than the ASC group (5.511 ± 1.022% and 5809 ± 1125) after 14 days of differentiation.

### 3.3. RT-PCR Results

**Figure 4:**
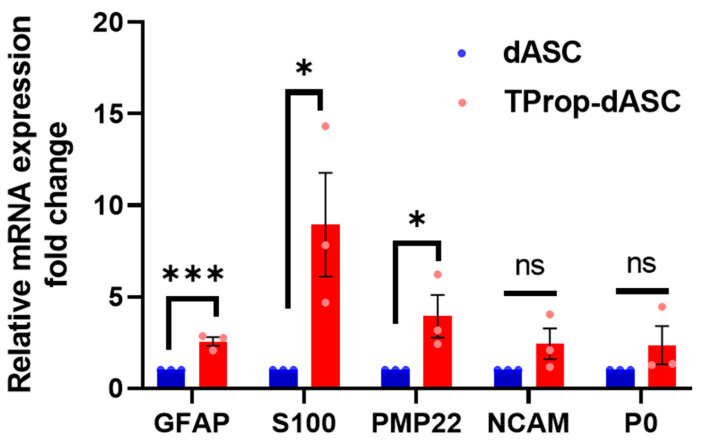
Transcript analysis of Schwann cell differentiation in ASCs. The RT-qPCR analysis for Schwann cell differentiation markers after 2 days of treatment with 50 µM TProp treatment (TProp-dASC) in differentiation medium showed significantly higher mRNA levels of GFAP, S100, and PMP22, but not for NCAM and P0. Statistical analysis of the RT-qPCR was carried out using the (2^−ΔΔCt^) method, which calculates the relative changes in mRNA levels normalized to an endogenous reference (GAPDH) relative to a calibrator (without analog treatment, dASC) that serves as the control group; the differences between treated and control samples were expressed as fold changes. * *p* < 0.05, *** *p* < 0.001 (*n* = 3, mean ± SEM; Student’s *t*-test; ns, no significant difference).

To determine the effects of TProp on ASC differentiation, RT-qPCR was used to measure transcript levels of SC markers (GFAP, S100, PMP22, NCAM, and P0) in differentiated ASCs two days post-induction. Transcript levels were normalized to GAPDH levels allowing a comparison of gene expression in the TProp-treated (TProp-dASC) and untreated groups (dASC). Significant upregulation of S100β, GFAP, and PMP22 was observed in the TProp-treated group (2.96 ± 0.34), compared to its untreated counterpart (1.47 ± 0.191) (*p* < 0.001) (Figure 4). Transcript levels of NCAM and P0 also trended higher in the TProp-dASCs group (2.21± 0.30), compared to the dASC group (1.72 ± 0.176), but this result was not statistically significant (*p* > 0.05).

### 3.4. Secretion of Neurotrophins

**Figure 5:**
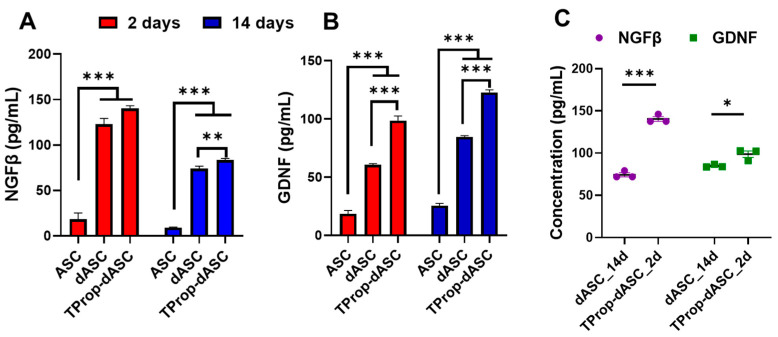
ELISA-based quantification of neurotrophic growth factors from ASC, dASC, and TProp-dASCs. Nerve growth factor (NGFβ, (**A**)) and glial-cell-derived neurotrophic factor (GDNF, (**B**)) secretion levels at 2 days (red bars) and 14 days (blue bars). (**C**) Comparison of NGFβ (purple circles) and GDNF (green squares) secretion between dASCs at 14 days and TProp-dASCs at 2 days. Both NGFβ and GDNF levels were increased in TProp-dASCs as compared with dASCs at either day 2 or day 14 time points. Furthermore, the levels of both of these factors were higher with TProp stimulation on day 2 than without TProp stimulation on day 14. Data are expressed as mean ± SEM. * *p* < 0.05, ** *p* < 0.01, and *** *p* < 0.001, one-way ANOVA with Tukey’s post-test for (**A**,**B**), and Student’s *t*-test for (**C**).

Concentrations of the NGFβ and GDNF secreted by ASCs, dASCs, and TProp-dASCs were determined by using ELISA. Overall, the concentrations of NGF and GDNF secreted by TProp-dASCs were higher than in the other two groups at both the 2- and 14-day time points (Figure 5A,B). GDNF secretion was higher at day 14 compared to day 2 in all groups whereas NGFβ levels showed a decrease of 39.34% and 40.71% for the dASC and TProp-dASC groups, respectively. For NGFβ, TProp-dASCs secreted significantly greater levels (*p* < 0.001) than either ASCs and dASCs at day 2 (140.45 ± 2.81 vs. 18.34 ± 6.86 and 122.91± 6.37 pg/mL). At day 14, TProp-dASCs secreted significantly higher levels of NGFβ than those observed from ASCs (83.61 ± 1.67 vs. 9.22 ± 0.61 pg/mL, *p* < 0.001), and dASCs (74.31 ± 2.46 pg/mL, *p* < 0.01). The levels of GDNF secreted by TProp-dASCs (98.71 ± 3.80 pg/mL at 2 days and 122.84 ± 2.20 pg/mL at 14 days) were higher than those from dASCs (60.71 ± 1.01 pg/mL, *p* < 0.001 at 2 days and 84.71 ± 1.01 pg/mL *p* < 0.01 at 14 days) and ASCs (18.57 ± 2.82 pg/mL, *p* < 0.001 at 2 days and 25.51 ± 2.12 pg/mL, *p* < 0.001 at 14 days). Moreover, Figure 5C showed that TProp-dASCs at 2 days secreted significantly higher levels of both NGFβ (140.45 ± 2.81 pg/mL, *p* < 0.001) and GDNF (98.71 ± 3.80 pg/mL, *p* < 0.05) than the levels of these proteins secreted by dASCs at 14 days (NGFβ 74.31 ± 2.46 pg/mL and GDNF 84.71 ± 1.01 pg/mL).

### 3.5. Wnt Signaling Activation

**Figure 6:**
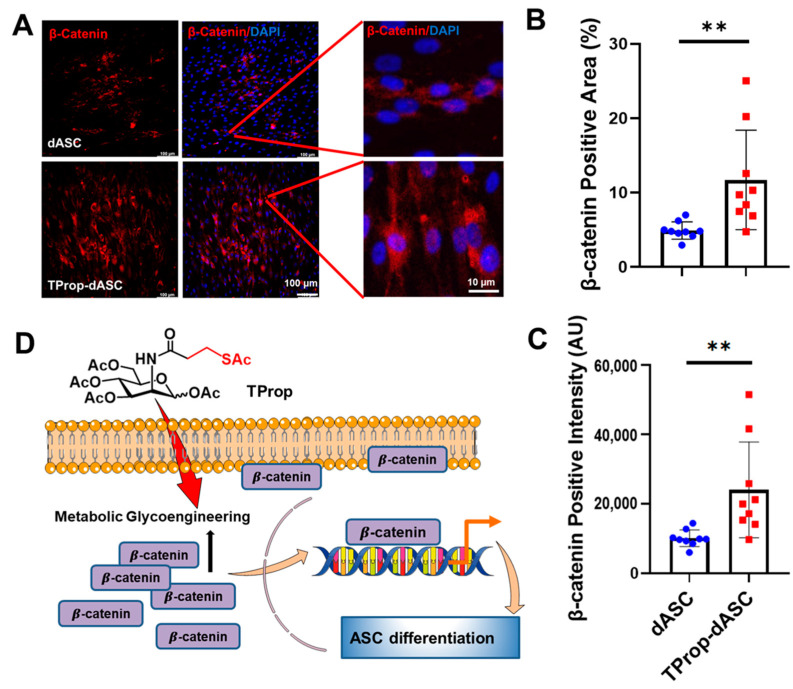
Wnt/β-catenin signaling activation. (**A**) Immunofluorescence staining of β-catenin (red) in dASCs and TProp-dASCs. Quantification of area fraction (**B**) and intensity of positive β-catenin cells (**C**). β-catenin was more highly expressed in TProp-dASCs compared to dASCs. ** *p* < 0.01, Student’s *t*-test. TProp-dASCs experienced significantly increased β-catenin signaling compared to dASCs. (**D**) Schematic representation of how TProp activates cell β-catenin for ASC differentiation. TProp enters ASCs through metabolic glycoengineering. Metabolic-treated dASC (TProp-dASC) caused a nearly twofold expression of β-catenin compared to dASC, which led to more β-catenin translocation to the nucleus. After translocating to the nucleus, β-catenin formed a complex to activate the transcription of downstream genes, thereby promoting the differentiation of ASCs into Schwann-cell-like cells.

Wnt signaling activation was monitored in ASCs that were treated, or not, with TProp. In these experiments, TProp-dASCs experienced significantly increased (*p* < 0.01) β-catenin expression (Figure 6A), specifically, levels increased approximately twofold (4.74 ± 2.23%) compared to dASC (2.93 ± 0.39%) (Figure 6B). Similarly, the β-catenin positive intensity of TProp-dASCs (24,048 ± 4586) was more than double (*p* < 0.01) that of untreated dASCs (10,104 ± 794.6, Figure 6C). A schematic representation of how TProp promotes the differentiation of dASCs into Schwann cell-like cells is shown in Figure 6D. This representation is based on experiments that showed that TProp-dASC caused a nearly twofold expression of β-catenin compared to dASC, which increased β-catenin translocation to the nucleus. After translocation to the nucleus, β-catenin presumably forms a multiprotein complex that activates the transcription of downstream genes, thereby promoting the differentiation of dASCs into Schwann cell-like cells via the Wnt signaling pathway.

## 4. Discussion

ASCs are being widely investigated both in vitro and in vivo as therapeutic cells able to aid in nerve regeneration due to their ability to differentiate into SC [13,24]. Despite extensive efforts to develop reliable methods to differentiate ASCs from SCs as therapeutic cells, current protocols generally take several weeks to complete the in vitro differentiation [13,14,40,41]. This study highlights a potential solution for the long in vitro preparation time required before in vivo implantation that hinders clinical translation efforts by reducing the differentiation timeframe of human ASCs from several weeks to two days. In particular, we compare a standard ASC differentiation protocol that requires two weeks with MGE-enabled differentiation using TProp that promotes the phenotypic development of human ASCs to an SC-like morphology, with greatly increased SC biomarkers expression and significantly secreted NGFβ and GDNF proteins level in two days. As a caveat, these protocols were optimized for rodent cultures. Consequently, because rat development is shorter than human development, longer differentiation protocols may be needed to achieve comparable differentiation in human cells.

To date, the majority of therapeutic applications of MGE have pursued cancer diagnostics or therapeutics [42,43]. In the current study, we pursue a less frequently explored aspect of MGE, which is its ability to alter cellular differentiation. For example, ManNProp promoted monocytic differentiation of HL60-cells [44]; contrarily, 3F-Neu5Ac inhibited osteogenic and adipogenic differentiation of mesenchymal stromal cells [45]. In previous studies, our team showed that thiolated ManNAc analogs can enhance the neural differentiation of hEBD cells and hNSCs [27,30], and recently, we showed that TProp suppresses adipogenic differentiation in hASCs without interfering with glial cell differentiation [30]. By introducing thiol-modified monosaccharide analogs into cellular metabolic pathways, resulting in biosynthetic incorporation of these non-natural sugars into the cell surface glycans, the previous studies indicated that MGE could manipulate cellular physiology and responses to promote ASC differentiation in ways that promise therapeutic value. In the current study, we have added a therapeutically relevant endpoint to these pioneering studies by demonstrating that MGE facilitates the rapid differentiation of ASCs into cells with an SC-like phenotype with the ability for enhanced secretion of neurotrophic factors.

Our findings, described in this report, demonstrated that conversion of the flattened fibroblast-like morphology of undifferentiated ASCs into spindle-shaped and bi/tripolar cells with elongated processes consistent with an SC-like morphology was enhanced by TProp (Figure 2). The differentiation percentage of ASCs into Schwann cells can vary depending on several factors such as culture conditions, cell source, and differentiation protocols. Several studies have reported successful differentiation of ASCs into Schwann cells with varying differentiation percentages from 40–80% [18,19]. Although ASCs can differentiate into Schwann cells under the published protocol, the use of specific sugar (TProp) stimulation can significantly enhance the differentiation percentage, from ~10% and 42% to 42% and 68% on day two and fourteen, respectively. In addition, the differentiation process is time-dependent, and longer induction periods lead to higher differentiation efficiencies. In particular, after 2 days of transdifferentiation, expression of the SC markers S100 and P75 significantly increased in TProp-treated cells compared to untreated cells. In addition, we observed MGE-dependent changes in morphology that were accompanied by expression changes at the protein (Figure 2 and Figure 3) and mRNA (Figure 4) levels consistent with SC differentiation, indicating the valuable role of MGE in this process. Mechanistically, we implicated β-catenin accumulation in TProp-dASCs (Figure 6), consistent with the involvement of Wnt signaling in previous studies with hEBD cells [27] and hNSCs [30]. The increase in β-catenin, just one of the multifactorial beneficial cell responses elicited by MGE for neural regeneration, includes its partitioning to the inner surface of the plasma membrane where it organizes cell adhesion molecules (consistent with the impact of TProp on cell adhesion [31]) as well as to the nucleus where it associates with transcription factors and directs neurogenic differentiation (Figure 5D).

ASCs represent a rich source of neurotrophic growth factors [46,47], which exert multiple beneficial effects for neurorestoration and tissue regeneration [48]. Our findings in the current report demonstrate that differentiation of human ASCs leads to upregulation of NGFβ and GDNF, consistent with previous studies [49,50]. NGFβ and GDNF were tested in this study because they are important for cell survival and neurite outgrowth during development and regeneration [51]. In particular, NGFβ is a key component in axonal regeneration and sprouting of primary sensory neurons [52], whereas GDNF promotes neuron survival and axon outgrowth [53]. NGFβ plays a significant role in promoting both sensory neuron survival and fiber outgrowth in peripheral sensory neurons [54], modulates the activity of immune cells, reduces inflammation, and promotes a favorable environment for nerve regeneration [55], which makes it a prominent factor in maintaining the integrity of the peripheral nervous system. Clinical studies have shown promising results for the use of NGFβ as a therapeutic agent for peripheral nerve injuries and conditions [56]. In our in vitro differentiation model, we evaluated the ability of MGE to modulate the production and release of neurotrophic factors in ASCs. For the first time, we demonstrated that TProp-dASCs produce and release higher levels of GDNF and NGFβ than those from dASCs (Figure 5). Accordingly, the ability of MGE to increase levels of secreted GDNF and NGFβ, and to do so rapidly (i.e., after two days), represents an important advance for efforts to use ASCs therapeutically for nerve regeneration. These findings represent the first evidence for the positive role of MGE in the modulation of neurotrophic factors expression and release in stem cells. One benefit of time-accelerated differentiation observed after two days is the ultimate shortening of treatment regimens, which will facilitate clinical translation of ASC-based therapies. A biological benefit is that although GDNF concentrations were maintained in both treatment groups in the 14-day differentiation protocol, NGFβ levels were down regulated during the longer two-week differentiation protocol. By incorporating MGE into the protocol, the high levels of NGFβ observed at the two-day time point in theory can be maintained for in vivo cell transplantation along with concomitant therapeutic benefits.

## 5. Conclusions

This study is the first to focus on promoting the differentiation of SCs from ASC through MGE. A key advance we report herein is that an MGE approach, specifically the use of the TProp analog, significantly reduces the transdifferentiation period of ASCs into SCs in two days compared to traditional ASC differentiation protocols that require a minimum of two weeks. Importantly, the SCs derived from MGE-treated ASCs were phenotypically similar to mature myelinating SCs and demonstrated the ability to secrete neurotrophins in vitro. In summary, MGE induction provides a novel strategy for obtaining precursor cells for use in nerve tissue engineering.

## Data Availability

All data presented within this study are available within the manuscript.
